# Come Together, Work Together, Achieve Together: Tensions in Leading Intersectoral Partnerships

**DOI:** 10.5334/ijic.9797

**Published:** 2025-04-08

**Authors:** Michelle L. A. Nelson, Niamh Lennox-Chhugani

**Affiliations:** 1 Lunenfeld-Tanenbaum Research Institute, Sinai Health, CA; 2 Institute of Health Policy, Management and Evaluation, University of Toronto, CA; 3 International Foundation for Integrated Care, NL

**Keywords:** intersectoral partnerships, collaboration, leadership, governance, collective impact

## Introduction

We frequently discuss the promise of collaboration across community sector organizations, public health providers, and private care providers that enable asset-driven services across organizational boundaries. Yet many organizational structures remain trapped within traditional siloed work. In pursuing intersectoral partnerships, leadership teams often face tensions between managing internal team processes and achieving collective impact across sectors. These challenges may include cultural and philosophical differences, varying approaches to achieving collective aims, and persistent disconnects in policy and funding environments. Through this editorial, we would like to explore these challenges and highlight potential strategies for leaders of intersectoral teams to “Come Together, Work Together, Achieve Together.” While there are many possible approaches to overcoming these challenges, we present some potentially underutilized or under-appreciated concepts for leading intersectoral partnerships.

## Challenge: Differing Organizational Norms, Practices, and Priorities

The importance of understanding members’ history, previous relationships, and perspectives early in forming new collaborative teams is well-established [1]. However, we also know that newly formed groups often rush to the performing stage of team formation, neglecting the essential phases of forming, storming, and norming, during which trust and understanding are built among partners [2]. This rush to action can result in superficial agreements lacking depth and commitment, ultimately jeopardizing the sustainability and effectiveness of integrated care initiatives. For example, some members may come from organizations with high levels of risk aversion, where decision-making processes are focused on maintaining control and minimizing uncertainty. This may pose a challenge in integrated care partnerships, which require higher risk-taking levels to foster innovation and adaptability [3,4]. This difference can create friction as integrated care leaders strive for health system innovation while respecting the varying perspectives of their collaborating organizations. Another example from practice: many collaborative teams we’ve worked with report the importance of representing diverse perspectives at decision-making tables. Yet, these teams also indicate that efforts to ensure inclusivity can hinder timely decision-making. Leadership groups may find themselves caught in a dilemma: the desire to authentically engage systemically underrepresented voices can prolong discussions that delay actions for which they are accountable, potentially impeding progress toward practical implementation.

## Rising to the Challenge

### Work with those we may disagree with

Moving away from ways of working that prioritize professional or sectoral perspectives requires collective commitment; this is not an operational shift that a single individual or small group can address alone. While convening partners from different sectors inevitably bring differing worldviews, conflict often lies at the heart of collaboration and presents growth opportunities. Key principles include embracing conflict—acknowledging that consensus isn’t necessary for progress, recognizing one’s role in the problem and solution, and maintaining momentum despite unpredictability. Perhaps success is better defined as the commitment to work together toward solutions rather than achieving definitive answers.

### Embrace Uncertainty

The desire for certainty is natural, particularly when managing resource-strained environments. However, integrated care systems are complex and demand a new leadership approach that tolerates uncertainty and embraces it. Embracing adaptive 21st-century leadership principles can help leaders navigate cross-sector collaboration, negotiate competing priorities, and maintain momentum under uncertain policy directions. Promoting psychological safety is crucial when managing cross-sectoral teams to work effectively within uncertainty while moving toward shared goals. These teams may even view uncertainty as a condition for true innovation.

## Challenge: Operational Strategies (Leadership and Governance)

Much has been written about moving beyond traditional leadership models, characterized by command-and-control structures, to modern frameworks that advocate for collaborative, inclusive, and distributed leadership, encouraging collective decision-making. Some thinkers have described this movement as a philosophical shift—from “Old Power,” rooted in exclusivity and hierarchy, to “New Power,” characterized by shared influence and participatory governance, reflecting broader societal changes in how healthcare leadership is perceived [5].

This is less about shifting power and more about changing the rules governing who controls resources and what resources matter in collaborative models [6,7]. The tension between technocracy and a more democratic approach to health and well-being creates friction as stakeholders negotiate power dynamics and governance structures that can either facilitate or hinder collaborative efforts. Embracing this tension is crucial for fostering an integrated care environment and prioritizing equity and shared responsibility.

Leadership practices combined with organizational versus system accountabilities present prominent challenges for integrated care efforts. While there is a growing need for shared accountability across the integrated care system to ensure collective goals are met, individual members remain responsible for achieving their home organizational objectives. This dual accountability creates ongoing tension in decision-making processes.

## Rising to the Challenge

### Collaborative or Co-Leadership

A common assumption is that shifting to a “community-driven” approach naturally evokes collaborative leadership models. However, intersectoral leaders repeatedly emphasize the need for clear guidelines that determine inclusion, authority over resources, and decision-making processes. The concept of co-leadership has recently garnered attention as an approach that promotes the representation of underrepresented perspectives [8].

Exploring the essential differences between co-leadership and collaborative leadership is warranted. While both emphasize teamwork and shared decision-making, co-leadership explicitly involves two or more individuals sharing designated formal leadership roles with equal authority. It’s a common practice in the private sector after mergers to support the perception of equal representation [9]. In contrast, collaborative leadership encompasses a broader group of stakeholders without the necessity of shared formal roles and may involve more decentralized decision-making. Co-leadership typically represents a long-term arrangement between designated leaders, while collaborative leadership can emerge for specific initiatives. Choosing an appropriate leadership approach should consider the longer-term aspirations of the collective.

The most critical skills of collaborative leaders encompass their individual characteristics, interpersonal skills, and group process abilities, followed by their strategic leadership and technical expertise [10]. This is the time to embrace leadership models that recruit individuals with the requisite interpersonal and facilitation skills to navigate complex collaborative environments. By cultivating emotional intelligence [11] and fostering collaboration, leadership groups can enhance their capacity to address challenges collectively, ultimately driving more effective outcomes in integrated care.

### Power Pooling

Many collaborative leadership councils describe power imbalances and routinely discuss the challenge of redistributing power from high-influence members to typically underrepresented individuals. A helpful concept to consider is “power pooling,” which was developed in electrical utility management [12]. Power pooling allows electricity producers to share resources and balance supply and demand over an extensive network, optimizing resource use, enhancing reliability, and sharing infrastructure. Applying this logic to intersectoral partnerships encourages us to consider power as a resource that members bring to collaborative efforts, recognizing that different partners contribute distinct types of power to the collaboration. Such an approach requires stable connections between collaborators and explicit work to establish new rules for what is “pooled,” how, for how long, and to whose benefit.

### Consider Mutuality of Interests rather than Conflict of Interest

The concept of ‘mutuality of interest’ contrasts with the widely accepted notion of ‘conflict of interest,’ particularly in collaborative governance contexts. Conflict of interest arises when individuals or organizations possess competing interests that may compromise impartiality, potentially eroding trust and undermining effective collaboration. Mutuality of interest emphasizes collaboration, shared goals, and interdependence among stakeholders, fostering relationships built on trust and the understanding that all parties benefit from collective efforts. Within this framework, collaborative decision-making fosters shared responsibility and transparency, resulting in mutually beneficial outcomes for all parties involved.

## Conclusion

While the path to effective intersectoral collaboration has challenges, these challenges also present opportunities for growth and innovation. By embracing the principles of “stretch collaboration,” leaders can navigate the inevitable conflicts that arise from diverse perspectives, transforming them into catalysts for deeper understanding and shared purpose.

As we confront the uncertainties inherent in integrated care systems, we must cultivate a culture of psychological safety that tolerates ambiguity and thrives within it. Embracing adaptive leadership principles allows us to respond dynamically to the evolving landscape of health and social care, ensuring our collective efforts remain focused on achieving equitable outcomes. Shifting from traditional, hierarchical decision-making models to collaborative frameworks that prioritize shared responsibility and inclusivity may help dismantle barriers that have historically impeded progress and foster an environment where all voices are valued, and collective impact is achieved.
